# Assessment of the 9p21.3 locus in severity of coronary artery disease in the presence and absence of type 2 diabetes

**DOI:** 10.1186/1471-2350-14-11

**Published:** 2013-01-23

**Authors:** Natalia V Rivera, Robert Carreras-Torres, Roberta Roncarati, Chiara Viviani-Anselmi, Francesca De Micco, Alessandra Mezzelani, Werner Koch, Petra Hoppmann, Adnan Kastrati, Alexandre FR Stewart, Li Chen, Robert Roberts, Lennart C Karssen, Najaf Amin, Valentina Trimarco, Raffaele Izzo, Guido Iaccarino, Gerolama Condorelli, Annibale A Puca, Paolo Pagnotta, Flavio Airoldi, Bruno Trimarco, Cornelia M van Duijn, Gianluigi Condorelli, Carlo Briguori

**Affiliations:** 1 IRCCS Multimedica, Via Fantoli 16/15, 20138, Milan, Italy; 2 Facultat de Biologia, Universitat de Barcelona, Barcelona, Spain; 3 Institute of Genetic and Biomedical Research (IRGB), National Research Council (CNR), Milan, Italy; 4 Institute of Biomedical Technologies (ITB), National Research Council (CNR), Milan, Italy; 5 Deutsches Herzzentrum München, Munich, Germany; 6 Ruddy Canadian Cardiovascular Genetics Centre, University of Ottawa Heart Institute, Ottawa, Canada; 7 Department of Genetic Epidemiology, Erasmus MC, Rotterdam, The Netherlands; 8 Department of Neuroscience, Federico II University, Federico II University Hospital, Naples, Italy; 9 Department of Clinical Medicine and Cardiovascular Sciences, Federico II University Hospital, Naples, Italy; 10 School of Medicine, University of Salerno, Baronissi, Salerno, Italy; 11 Department of Biology, Cellular and Molecular Pathology, University Federico II, Naples, Italy; 12 Humanitas Clinical and Research Center, Milan, Rozzano, Italy; 13 Laboratory of Interventional Cardiology, Department of Cardiology, Clinica Meditterranea of Naples, Via Orazio 2, 80121, Naples, Italy

**Keywords:** Severity of CAD, Coronary artery disease, Diabetes mellitus, T2D, 9p21.3, Genetics, Single nucleotide polymorphism

## Abstract

**Background:**

The 9p21.3 locus is strongly associated with the risk of coronary artery disease (CAD) and with type 2 diabetes (T2D). We investigated the association of 9p21.3 variants with severity of CAD (defined by the number of vessel diseased [VD]) in the presence and absence of T2D.

**Methods:**

We tested 11 9p21.3-variants for association in a white Italian study (N = 2,908), and carried out replication in 2 independent white populations, a German study (N = 2,028) and a Canadian Study (N=950). SNP association and permutation analyses were conducted.

**Results:**

We identified *two* 9p21.3-variants, rs4977574 (*P* < 4×10^-4^) and rs2383207 (*P* < 1.5×10^-3^) that were associated with severity of CAD in subjects without T2D. Association of rs4977574 with severity of CAD was confirmed in the Canadian Study. Results from subgroup analysis among patients with T2D showed an interaction between rs10738610 and T2D with *P* = 4.82×10^-2^. Further investigation showed that rs10738610 (*P* < 1.99×10^-2^) was found to be significantly associated with severity of CAD in subjects with T2D.

**Conclusions:**

The 9p21.3 locus is significantly associated with severity of CAD. The number of associations of 9p21.3 variants with severity of CAD is variable to the presence and absence of T2D. In a CAD-susceptible region of 115 kb, there is only one variant associated with the severity of coronary vessel disease in the presence of type 2 diabetes.

## Background

Coronary artery disease (CAD) concomitant with type 2 diabetes (T2D) is the leading cause of cardiovascular (CV) morbidity and mortality. Severity of CAD is higher in diabetics than in non-diabetics, often presenting itself as a 3-vessel disease with a more diffuse coronary artery involvement. Thus, diabetics have higher risks for complications frequently leading to death [1].

The combination of genetics, intermediate phenotypes, and environmental factors (such life-style) play a major role in the pathogenesis of CAD and T2D, including determining the conditions and stability of these diseases. The genetic architectures of CAD and T2D are dependent on multiple genetic and environmental triggers that may overlap or may be specific to each disease. The estimated heritability of CAD ranges from 30 to 60% [2,3], whereas of T2D is from 40 to 90% [4,5].

To date, the 9p21.3 locus is the most consistently replicated genetic locus for CAD [6-11] and T2D [12-14]. The association with this locus is independent of cardiovascular risk factors, including age, gender, obesity, smoking, hypertension, and hyperlipidemia. Moreover, the 9p21.3 locus is phenotypically heterogeneous, since it is also associated with various phenotypes, including myocardial infarction [15], arterial stiffness [16], aortic aneurysm [17], ischemic stroke [18,19], glioma [20], and malignant melanoma [21]. The role of the susceptible region for CAD and T2D at the 9p21.3 locus, a region located on two adjacent haplotype blocks, a 44-kb (from rs10116277 to rs1333049) and a 4-kb block (from rs10965243 to rs10757283) [22], has been a major question of research for determining its implication in the pathogenesis of these diseases. Clearly, identifying genetic variants associated with severity of CAD in the presence and absence of T2D is crucial for understanding to what extent genetic variations at the 9p21.3 locus interplay in the pathogenesis of these conditions.

This study was undertaken to investigate the association of 9p21.3-variants with severity of CAD in patients without and with T2D. Thus, first, we sought to identify whether 9p21.3 variants have a role in determining the severity of CAD; and second, we assessed the impact of T2D in the associations with severity of CAD.

## Methods

### Subjects and phenotype characterization

Participants were of white European ancestry. All studies had protocols approved by local institutional boards. Participants provided written consent and gave permission to use their DNA for research purposes. Baseline characteristics for all participating studies are presented in Additional file 1: Table S1. Baseline measures of clinical and demographic characteristics were measured at each medical facility at the time of the participants’ enrollment in the study.

In all participating studies, severity of CAD was defined according to angiograms showing number of vessels disease ≥ 1 (namely, 1-vessel, 2-vessel, and 3-vessel disease) with > 50% lumen diameter stenosis and in accordance with clinical standards and guidelines for coronary angiography. T2D was defined as treatment with insulin or oral hypoglycemic drugs, or as elevated (> 126 mg/dL or > 7.0 mmol/L) levels of fasting, non-stressed blood glucose on at least two separate occasions in conjunction with adhering to ongoing dietary measures to control glucose level. Early-onset CAD was defined as onset ≤ 55 years of age and late-onset CAD as otherwise. Type 1 diabetics were excluded in all studies.

The Italian study consists of 2,908 subjects (67.2% male) aged 20 to 90 years diagnosed with and without severity of CAD. Specifically, 1,696 subjects were recruited from both the Clinica Mediterranea in Naples and the Multimedica Hospital in Milan and 1,212 were recruited from the Campania Salute (CS) Network at the University Federico II in Naples. Subjects from the Clinica Mediterranea and the Multimedica Hospital were scheduled for first-time elective coronary angiography because of clinical suspicion and/or diagnoses of CAD. Herein, an angiogram was called “normal” (or “unaffected without severity of CAD”) if it showed number of vessels <1 and smooth lumens with stenosis < 50%, whereas if it showed otherwise, the angiogram was called “affected with severity of CAD” and was further characterized by objective coronary angiography and in accordance with the ACC/AHA guidelines for coronary angiography [23]. Within the 1,696 subjects, 1,541 were “affected with severity of CAD” and 155 were “unaffected without severity of CAD”. The remaining 1,212 subjects were drawn from the Campania Salute (CS) Network, an open registry that collects information from a network of general practitioners and hospitals linked with a coordinating center (University Federico II in Naples). These subjects were used as “controls” (or “unaffected without severity of CAD”) and were selected based on their medical history and absent diagnosis for cardiovascular disease predefined by the exclusion criteria of the open registry study design. Exclusion criteria of the CS Network included history of previous myocardial infarction or angina or procedures of coronary revascularization, stroke, or transitory ischemic attack, congestive heart failure, and chronic kidney disease (defined as GFR < 30 ml/min per 1.73 m^2^). Additionally, participants enrolled in the CS network were subjected to an active follow-up program including periodically cardiac and carotid ultrasounds aimed to the prevention and the reduction of cardiovascular events in a population of hypertensives. Patients who presented signs of preclinical atherosclerosis or CAD were subjected to further exams, including coronary angiography. Further details on the design of the CS Network can be found elsewhere [24].

Combining all subjects from the three centers, we had a total sample size of 2,908 participants. Within this cohort, 19% of participants were diabetics. Additionally, 15% of patients had early-onset CAD and 31% had late-onset CAD according to available data records.

The German study included 2,028 subjects (78% males) aged 27 to 92 years characterized with ischemic symptoms or evidence of myocardial ischemia in the presence of 50% *de novo* stenosis located in native coronary vessels. Specifically, 1,917 patients were “affected with severity of CAD” and 111 were “unaffected without severity of CAD”. Severity of vessel disease was recorded as qualitative morphological lesion conditions by means of standard criteria. Baseline, post-procedural, and follow-up angiograms were digitally recorded and assessed off-line in the quantitative angiographic core laboratory (ISAR Center) with an automated edge-detection system (CMS version 7.1; MEDIS Imaging Systems, Leiden, the Netherlands) by two independent operators. Herein, 28% of subjects were diabetics. Further details of this study are described elsewhere [25].

The Canadian study was part of the Ottawa Heart Genomics Study [26] and consisted of 950 subjects (71% males) aged < 55 (males) and < 65 (female) with at least one epicardial stenosis > 50% who were considered as early onset cases of CAD. All subjects were “affected with severity of CAD”. The coronary angiograms were assessed by two independent angiographers blind to the results of the genotype. For a vessel to be scored, stenosis > 50% had to be noted in an epicardial coronary vessel of interest or in one of its major branches. In the event of discordance on the number of vessel scored between two reviewers, angiograms were scored by a third independent reviewer. Modified Gensini and modified Duke coronary scores were derived in subjects who were not previously bypassed. Subjects who had previously undergone PCI for a lesion for which the degree of stenosis was not known were not analyzed. Herein, all subjects were non-diabetics. Exclusion of diabetic patients was part of the study design and their exclusion criteria of the Ottawa Heart Genomics Study. Further details of this study and subject characteristics are described in detailed elsewhere [27].

### Specimen collection, SNP selection, and genotyping

A blood sample was drawn from each participant during the regular routing examination for coronary artery angiogram. Genomic DNA was extracted from peripheral blood lymphocytes using the DNA Qiamp Midi kit (QIAGEN Inc. Valencia, CA) and genotyped accordingly.

In the Italian study, 11 SNPs (rs7044859, rs10965215, rs564398, rs7865618, rs10116277, rs4977574, rs2383207, rs10738610, rs10757278, rs1333049, rs10811661) were selected from published genome-wide association studies (GWAS) of CAD [9,11,15] and T2D [13,14] - that is, 7 SNPs were chosen at *P* = 1×10^-8^ and 4 SNPs at *P* < 1×10^-6^. Herein, genotyping was carried out in two stages; in the first stage 1,696 subjects (from Clinica Mediterranea and Multimedica Hospital) were genotyped using TaqMan® Pre-Designed SNP Genotyping Assays (Applied Biosystems), whereas in the second stage 1,212 subjects (from the CS Network) were genotyped with Illumina Human1M-Duo BeadChip array. In the array, 7 of 11 selected SNPs were present and thus extracted after quality control filtering, which included minor allele frequency (MAF) ≤1%, Hardy-Weinberg equilibrium (HWE) *P* < 1×10^-6^, and SNP and subject call rates ≤ 97%. The missing SNPs (rs10116277, rs10738610, rs10757278, rs1333049) were imputed with reference to HapMap CEU release 22 build 36 using the maximum likelihood method implemented in MACH (version 1.0.15). Further details on genotyping and imputation are given in Additional file 1: Table S3. Genotypes of Italian participants were pooled together, resulting in 2,908 subjects and 11 SNPs to analyze.

In the replication stage: in the German study, SNP rs1333049 was available for replication and selection for studying the severity of CAD. Genotyping of this SNP in 2,028 subjects was performed using the TaqMan allelic discrimination assay. Further details of genotyping and quality control were available elsewhere [25], whereas in the Canadian study, SNP rs4977574 was available for replication and selection for studying the severity of CAD. This SNP was extracted from the Affymetrix (Santa Clara, California) 500 K array after GWAS quality control in 950 subjects. Details of genotyping and quality control were available elsewhere [27].

Observed genotypes and allele counts for all participating studies are shown in Additional file 1: Table S4.

### Statistical analysis

#### Statistical analysis within studies

Cardiovascular risk factors (i.e., LDL-cholesterol, HDL-cholesterol, triglycerides, type 2 diabetes mellitus, hypertension, etc.) obtained at baseline were analyzed using SPSS (version 17, SPSS, Chicago, Illinois, USA) and expressed as mean ± SD. Continuous variables were presented as means ± SD and categorical variables as absolute numbers and proportions (%) with one-way analysis of variance and chi-squared tests. These tests were also used to determine difference by genotype for some 9p21.3 variants. Clinical variables deviating from normal distribution were transformed by taking the natural logarithm of their value. Additional quantifying scores for severity of CAD, such as the Gensini score [28] (or modified Gensini [29]), the CAD Prognostic Index (also called the Duke score) [30], modified Duke [31], Dahlen score [32], and percent stenosis diameter (defined by the modified ACC/AHA classification) were assessed with linear regression. Hardy-Weinberg equilibrium (HWE) test was carried out for each SNP in the control group. SNPs with HWE *P* < 5×10^-2^ were excluded from the analysis. For the analysis of genetic data, we used the GenABEL [33] library of the R package software.

#### Association analysis

In the Italian study, we conducted association analyses using linear regression method under an additive genetic model and executed the analyses in two stages; first, an association analysis on the 11 SNPs regressed on severity of CAD (kept as a quantitative variable) using age, sex, and T2D as covariates; and second, an association analysis stratified by T2D on the 11 SNPs regressed on severity of CAD using age and sex as covariates after performing interaction tests between 9p21.3 SNPs and T2D. In both analytical stages, we supported nominal *P* value significance with empirical *P* values (i.e., *P*_*emp*_), which were computed by performing 10,000 permutations on the 9p21.3 region that included the 11 SNPs tested. Association and permutation analyses were performed using methods implemented in the GenABEL [33] library of the R software.

In the replication stage: in the German study, association analysis was carried out using linear regression method on severity of CAD under an additive model and adjusted for age, sex, and T2D. Additionally, association in stratified groups by T2D was performed after testing for interaction between rs1333049 and T2D. Association was performed using GenABEL package [33]. In the Canadian study, the association analysis was conducted using a logistic regression method. Severity of CAD in the early-onset CAD group (as defined by the study design) was dichotomized in two vessel disease groups: (a) 1-VD versus 2- and 3-VD, and (b) 1- and 2-VD versus 3-VD. Logistic regression was performed under an additive genetic model, adjusted for age and sex.

## Results

### Study samples

The characteristics of the patients participating in these studies are shown in Additional file 1: Table S1. Additionally, baseline characteristics by 9p21.3 genotypes for severity of CAD are shown in Additional file 1: Table S2. In the Italian study, 17% of participants had single-vessel (1VD), 20% had double-vessel (2VD), 16% had triple-vessel (3VD); the remaining 47% had normal coronary arteries or a stenosis diameter of < 50%. Furthermore, 64.5% had severity of CAD without T2D and the remaining percent had severity of CAD with T2D. In the German study, 83.4% of participants had 1VD, 11% had 2VD, and 5.5% had normal coronary vessels. Herein, 68.5% of participants had severity of CAD without T2D and the remaining percent had severe CAD with T2D. In the Canadian study, 30.1% of participants had 1VD, 32.9% had 2VD, and 36.9% had 3VD. In the early onset CAD, 19.8% of participants had 3VD and 35.6% had 1VD and 2VD. In the late onset CAD, 17.1% of participants had 3VD and 27.5% had 1VD and 2VD. Within this study, all participants had severity of CAD without T2D. Results (Table 1) of the association analysis performed on the 11 SNPs in the 2,908 Italian subjects showed that two SNPs (rs4977574 and rs2383207) in high LD (*r*^*2*^ > 0.93) were significantly (*P*_emp_ < 0.05) associated with severity of CAD after correcting for multiple testing. Results from testing interaction between 9p21.3 SNPs and T2D are shown in Additional file 1: Table S5. Results (Table 2) of the association analysis stratified by T2D revealed that the association between these two SNPs and severity of CAD continued to be present in subjects without and with T2D. Additionally, a third SNP rs10738610 was found to be significantly (*P*_emp_ < 1.99×10^-2^) associated with severity of CAD in the subjects with T2D. This SNP was imputed in 1,212 controls and had an imputation quality of *R*^*2*^ = 0.96. Imputation quality of imputed SNPs is shown in Additional file 1: Table S3. The linkage disequilibrium (LD, denoted by *r*^*2*^) among the 11 SNPs is shown in Additional file 1: Table S7.

**Table 1:** Association results derived from linear regression between 11 9p21.3-variants and severity of disease (defined as quantitative trait) in the Italian study (N=2,908)

**SNP**	**Position**	**Alleles**	**MAF**	**Beta**	**s.e.**	***P***	***P***_***emp***_
rs7044859	22,008,781	***A****/T*	0.463	0.015	0.025	5.46E-01	9.87E-01
rs10965215	22,019,445	***A****/G*	0.435	0.029	0.024	2.31E-01	7.38E-01
rs564398	22,019,547	*C/****T***	0.301	0.060	0.026	**2.44E-02**	1.34E-01
rs7865618	22,021,005	***A****/G*	0.304	0.063	0.026	**1.66E-02**	9.44E-02
rs10116277	22,071,397	*G/****T***	0.390	0.069	0.026	**9.14E-03**	5.90E-02
rs4977574	22,088,574	*A/****G***	0.398	0.107	0.025	**1.75E-05**	**4.00E-04**
rs2383207	22,105,959	*A/****G***	0.360	0.100	0.027	**1.98E-04**	**1.50E-03**
rs10738610	22,113,766	*A/****C***	0.386	0.070	0.026	**8.36E-03**	5.30E-02
rs10757278	22,114,477	*A/****G***	0.416	0.036	0.025	1.51E-01	5.62E-01
rs1333049	22,115,503	***C****/G*	0.421	0.041	0.025	9.52E-02	4.01E-01
rs10811661	22,124,094	*C/****T***	0.206	0.033	0.031	2.88E-01	8.22E-01

Boldface allele denotes coded allele; MAF, minor allele frequency; Genetic effects (betas) adjusted according to *severity of CAD ~ age+sex+T2D + SNP.* Boldface *P* value denotes statistical nominal significance <0.05; *P*_*emp*_ denotes empirical significance derived from 10,000 permutations on the 9p21.3 region including the 11 SNPs tested.

**Table 2:** Association results derived from linear regression between 11 9p21.3-variants and severity of disease (defined as quantitative trait) in the Italian study (N=2,908) stratified by T2D

					**Without T2D (N=2,343)**				**With T2D (N=565)**			
**SNP**	**Position**	**Alleles**	**MAF**	***P***_***int***_	**Beta**	**s.e.**	***P***	***P***_***emp***_	**Beta**	**s.e.**	***P***	***P***_***emp***_
rs7044859	22,008781	**A**/T	0.463	7.14E-01	0.011	0.028	6.94E-01	9.99E-01	0.024	0.054	6.52E-01	9.99E-01
rs10965215	22,019,445	**A**/G	0.435	6.82E-01	0.023	0.027	3.93E-01	9.27E-01	0.053	0.052	3.09E-01	8.79E-01
rs564398	22,019,547	C/**T**	0.301	7.66E-01	0.063	0.030	**3.32E-02**	1.70E-01	0.032	0.058	5.79E-01	9.96E-01
rs7865618	22,021,005	**A**/G	0.304	-	0.068	0.029	**1.99E-02**	1.09E-01	0.030	0.059	6.15E-01	9.98E-01
rs10116277	22,071,397	G/**T**	0.390	9.83E-02	0.054	0.029	6.01E-02	2.78E-01	0.138	0.067	**4.07E-02**	2.24E-01
rs4977574	22,088,574	A/**G**	0.398	9.53E-02	0.087	0.028	**1.72E-03**	**1.17E-02**	0.177	0.055	**1.21E-03**	**9.40E-03**
rs2383207	22,105,959	A/**G**	0.360	5.33E-02	0.081	0.029	**5.37E-03**	**3.39E-02**	0.198	0.069	**4.52E-03**	**2.91E-02**
rs10738610	22,113,766	A/**C**	0.386	**4.28E-02**	0.045	0.029	1.14E-01	4.53E-01	0.201	0.067	**2.92E-03**	**1.99E-02**
rs10757278	22,114,477	A/**G**	0.416	2.44E-01	0.019	0.028	5.01E-01	9.77E-01	0.107	0.054	**4.61E-02**	2.47E-01
rs1333049	22,115,503	**C**/G	0.421	6.44E-01	0.028	0.028	3.25E-01	8.60E-01	0.099	0.053	6.26E-02	3.16E-01
rs10811661	22,124,094	C/**T**	0.206	9.85E-01	0.053	0.035	1.26E-01	4.86E-01	0.051	0.068	4.56E-01	9.74E-01

MAF, minor allele frequency; *P*_int_, p-value from interaction tests by T2D as shown in Additional file 1**:** Table S5; Genetic effects (betas) adjusted according to model: *severity of CAD ~ age+sex+SNP;* Boldface allele denotes coded allele; Boldface *P* value denotes statistical nominal significance <0.05; *P*_*emp*_ denotes empirical significance derived from 10,000 permutations on the 9p21.3 region including the 11 SNPs tested.

Association tests employing other quantifying angiographic scores, including Duke scores, Dahlen scores, and percent diameter stenosis showed significant evidence of association for rs4977574 and rs2383207 with severity of CAD, as shown in Additional file 1: Table S6.

Results from the replication stage are shown in Tables 3 and 4. In the Canadian study, we observed an association between rs4977574 and severity of CAD in the early-onset CAD patients without T2D, which validated our result for this SNP in this particular group. No genotype data for this SNP was available in the late-onset group. In the German study, we observed no association between rs1333049 and severity of CAD. In addition, no association was detected in the stratified groups by T2D. This finding is in agreement with what we observed for this SNP.

**Table 3:** Replication results; Association results derived from logistic regression between rs4977574 and severity of CAD without T2D in Italian (N=2,343) and Canadian studies (N=950)

		**EO CAD**	
		**OR (95% CI)**	***P***
**3VD vs. (2VD+1VD)**			
rs4977574	Italian	1.734 (1.142-2.633)	**9.77E-03**
	Canadian	1.381 (1.124-1.698)	**2.14E-03**
**1VD vs. (2VD+3VD)**			
rs4977574	Italian	0.721 (0.542-0.958)	**2.42E-02**
	Canadian	0.648 (0.527-0.795)	**3.28E-05**

Odds ratio (OR) adjusted for age and sex; 05 CI, 95% confidence interval of OR; Boldface *P* denotes nominal P value significance <0.05; EO, early-onset CAD; 1VD=1-vessel disease; 2VD=2-vessel disease; 3VD=3-vessel disease.

**Table 4:** Replication results; Association results derived from linear regression between rs1333049 and severity of CAD in the German study (N=2,028)

**SNP**	**Position**	**Alleles**^**†**^	**MAF**	**Beta**	**s.e.**	***P***	***P***_***emp***_
rs1333049	22,115,503	**C**/G	0.421	0.0119	0.012	3.45E-01	3.44E-01

*Beta adjusted for age and sex; ^†^Boldface allele denotes coded allele; *P* denotes nominal significance; *P*_*emp*_ denotes empirical significance derived from 10,000 permutations.

## Discussion

In this study, our aim was to investigate the association between 9p21.3-variants and the severity of CAD in the presence and absence of T2D. Results from our analysis showed that *two* 9p21.3-variants (rs4977574 and rs2383207) from the known CAD-associated haplotype, are also associated with severity of CAD. We also showed that these variants are associated with severity of CAD in subjects with and without T2D. Additionally, a third SNP rs10738610, located inside the same CAD-associated haplotype, was found to be significantly associated with severity of CAD in subjects with T2D. The significance of the association for this SNP in the pooled analysis was slightly above the threshold; however, this heightened significance was strengthened after stratification by T2D. Association of rs4977574 with severity of CAD was confirmed in an independent study in subjects without T2D. The results of the association for SNP rs1333049, which is a well-documented variant in the association with incidence of CAD and is in high LD with rs494457 (*r*^*2*^ = 0.89), rs2383207 (*r*^*2*^ = 0.89), and rs10738610 (*r*^*2*^ = 0.93), showed no association with severity of CAD in the Italian and German studies. However, Dandona et al. [34] in the Canadian study demonstrated that this SNP predicted the severity of coronary atheromatous burden in patients without T2D.

From our results, we hypothesized that although the 9p21.3 locus does not contain any genes, there are two different pathological mechanisms harboring the same haplotype, one that shares a common genetic platform between molecular mechanisms of CAD and T2D and the other, an independent one that is linked to the pathogenesis of CAD alone. Additionally, disagreement on the associations with severity of CAD of well-documented 9p21.3 variants associated with CAD variants may be due to various factors, including phenotype heterogeneity and difference on risk-allele frequencies among participating studies. The mechanisms of severity of CAD and T2D in patients with acute coronary syndrome (ACS) are complex, and they are not so clear at present. While many genetic studies, including GWAS, for CAD and T2D, have identified the 9p21.3 locus as a common risk region (determined by *two* adjacent haplotypes), there are no functional variants (non-coding variants) within this region. Therefore, the link between the 9p21.3 locus and the genetic backbones of these two diseases still remains poorly understood. The 9p21.3 locus lies in proximity to *CDKN2A* and *CDKN2B*. These cyclin-dependent kinase inhibitors (CDKNs) generate several transcript variants that are involved in diverse biological processes, including cell proliferation, cellular senescence, cell cycle arrest, induction of apoptosis, and activation of caspase activity, to name a few [35,36]. Some of these biological processes are presumably implicated in the pathogenesis of atherosclerosis as well as in the other complex phenotypes (i.e., myocardial infarction, aortic aneurysm, ischemic stroke, T2D, glioma, and malignant melanoma). Recent works have speculated that the pathogenic role of the 9p21.3 locus in atherosclerosis is influenced by gene expression [37,38] and a transcriptional and translational control mechanism presumably caused by *cis*- and *trans*-acting elements located in the vicinity of this region. Yet, no c*is-* and/or *tran*s-acting mechanisms have been identified. Other works have elaborated many discussions about the analysis of the 9p21.3 locus and the appropriate phenotypic characterization. Thus, genotypic variability among 9p21.3 variants may be due to wrong phenotyping rather than sequence variations influenced by gene expression. Moreover, it seems that the significance of variants found at this locus is changeable to the phenotype of interest, its severity, and the presence of combined risk and environmental factors that correlated with the phenotype.

In our study, we have a well-characterized phenotype for severity of CAD in subjects with and without T2D. We performed association analyses in three different white populations of European ancestry, totaling 5,886 subjects. This enabled us to study the relationship between the 9p21.3 locus, including CAD- and T2D-associated variants, and the severity of CAD in subjects with and without T2D. Moreover, to reduce phenotype-genotype bias, we used additional scoring methods for assessing extension of CAD. Then, we conducted association analyses following two approaches for assessing the relationship between severity of CAD and 9p21.3-variants. Our finding showed (1) that rs4977574 and rs2383207 at the 9p21.3 locus are associated with severity of CAD regardless of T2D status and (2) that rs10738610 is associated with severity of CAD comorbid with T2D.

This study has limitations: (1) in order to characterize the 9p21.3 locus more effectively in subjects with severity of CAD and T2D, more variants need to be included in the analysis and hence tested for association with this phenotype; (2) observed differences among genetic effects of 9p21.3 variants across populations may be due to the quantitative method in which angiographic scores were calculated in each participating study; (3) lack of availability of variants rs2383207 and rs10738610 in replication stage demands validation of their associations with severity of CAD in other white population studies with similar characteristics; (4) phenotype heterogeneity among participating studies is likely to be present since each study was previously designed to answer its own research question and had predefined inclusion and exclusion criteria; (5) In the Italian study, although we cannot discard the presence of atherosclerosis in the 1,212 controls, preliminary testing between 9p21.3 variants and incidence of CAD obtained from follow-up records showed no association with CAD; (6) lack of availability of 3-vessel disease subjects in the German study may had underestimated the genetic effect of SNP rs1333049 attained in the replication stage; and (7) lack of availability of genotype data of SNP rs497754 in late-onset CAD subjects with severity of CAD in the Canadian study requires a replication in another cohort with similar characteristics as to strengthen the significance of this SNP with severity of CAD regardless on the onset of the disease.

## Conclusions

The 9p21.3 locus is significantly associated with severity of CAD. The number of associations of 9p21.3 variants with severity of CAD is variable to the presence and absence of T2D. In a CAD-susceptible region of 115 kb, there is only one variant associated with the severity of coronary vessel disease in the presence of type 2 diabetes.

## Abbreviations

CABG: Coronary artery bypass graft surgery; CAD: Coronary artery disease; T2D: Type 2 diabetes mellitus; eGFR: Estimated glomerular filtration rate; GWAS: Genome-wide association study; HDL-C: High density lipoprotein cholesterol; HWE: Hardy-Weinberg equilibrium; LD: Linkage disequilibrium; LDL-C: Low density lipoprotein cholesterol; P_emp_: Empirical P-value; SNP: Single nucleotide polymorphism; kb: kilo base pairs.

## Competing interests

The authors declare that they have no competing interests.

## Authors’ contributions

Conceived and designed the experiments: CB, NVR, GC, RR, and CVA. Performed the clinical work: CB and FA. Performed the biological experiments: RR, CVA, and AM. Analyzed the data: NVR, FDM, LCK, and RCT. Performed study in German population: WK, PH, and AK. Performed study in Canadian population: AFRS, LC, RR. Contributed reagents/materials/analysis tools: RR, CVA, AM, AAP, NVR, P.P. and FDM. Wrote the paper: NVR, RCT, CMVD, NA, AFRS , CB, and GC. Other: drafting article NVR, CB, GC, RR, and CVA. All authors read and approved the final manuscript.

## Pre-publication history

The pre-publication history for this paper can be accessed here:

http://www.biomedcentral.com/1471-2350/14/11/prepub

## Supplementary Material

Additional file 1 **Table S1.** Demographics and comorbidities of participants. A.Italian study (N=2,908). B.German study (N=2,028). C.Canadian study (N=950). **Table S2.** Patient characteristics by 9p21.3 (rs4977574, rs2383207, and rs10738610) genotypes for severity of CAD. **Table S3.** Genotyping information of genotyped and imputed SNP in the Italian study (N=2,908). **Table S4.** A.Observed genotypes and allele counts (proportions) in the Italian study (N=2,908). B.Observed genotypes and allele counts (proportions) in the German study (N=2,028). C. Observed genotypes and allele counts (proportions) in the Canadian study (N=950). **Table S5.** Association results derived from linear regression between severity of CAD and 9p21.3 SNPs with interaction term (T2D * SNP) performed in the Italian study (N=2,908). **Table S6.** Association results of rs4977574, rs2383207, and rs10738610 with other quantifying scores for severity of CAD in the Italian (N=2,908) study. **Table S7.** Linkage disequilibrium (LD) among the 11 9p21.3 SNPs in the Italian study (N=2,908). Click here for file
