# Supplementary material for: Assessment of the 9p21.3 locus in severity of coronary artery disease in the presence and absence of type 2 diabetes
Source: BMC Med Genet. 2013 Jan 23;14:11. doi: 10.1186/1471-2350-14-11 (PMC3556499; doi:10.1186/1471-2350-14-11)
Supplement: Additional file 1 — Table S1. Demographics and comorbidities of participants. A.Italian study (N=2,908). B.German study (N=2,028). C.Canadian study (N=950). Table S2. Patient characteristics by 9p21.3 (rs4977574, rs2383207, and rs10738610) genotypes for severity of CAD. Table S3. Genotyping information of genotyped and imputed SNP in the Italian study (N=2,908). Table S4. A.Observed genotypes and allele counts (proportions) in the Italian study (N=2,908). B.Observed genotypes and allele counts (proportions) in the German study (N=2,028). C. Observed genotypes and allele counts (proportions) in the Canadian study (N=950). Table S5. Association results derived from linear regression between severity of CAD and 9p21.3 SNPs with interaction term (T2D * SNP) performed in the Italian study (N=2,908). Table S6. Association results of rs4977574, rs2383207, and rs10738610 with other quantifying scores for severity of CAD in the Italian (N=2,908) study. Table S7. Linkage disequilibrium (LD) among the 11 9p21.3 SNPs in the Italian study (N=2,908). [file 1471-2350-14-11-S1.doc]

**SUPPLEMENTAL MATERIAL**

**The role of 9p21.3 locus in severity of coronary artery disease in present and absence of diabetes mellitus**

Natalia V Rivera, MSc, PhD^1,2^; Robert Carreras-Torres, MSc^3^; Roberta Roncarati, PhD^1,4^; Chiara Viviani Anselmi, MSc^1^; Francesca De Micco, PhD^1^; Alessandra Mezzelani, PhD^5^; Werner Koch, MD, PhD^6^; Petra Hoppmann, MD^6^; Adnan Kastrati, MD^6^; Alexandre F.R. Stewart, PhD^7^; Li Chen, MSc^7^; Robert Roberts, MD, FRCPC, MACC ^7^; Lennart C Karssen, PhD^2^; Najaf Amin, MSc, PhD^2^; Valentina Trimarco, PhD^8^; Raffaele Izzo, MD^9^; Guido Iccarino, MD, PhD^10^; Gerolama Condorelli, MD, PhD^11^; Annibale A. Puca, MD, PhD^1^; Paolo Pagnotta, MD^12^; Flavio Airoldi, MD^1^; Bruno Trimarco, MD, PhD^9^; Cornelia M van Duijn, PhD^2^,; Gianluigi Condorelli, MD, PhD^4,13+^; and Carlo Briguori, MD, PhD^14+^

**Table of contents:**

**SUPPLEMENTARY TABLES Page 3**

**SUPPLEMENTARY TABLES**

**Supplementary Table 1** Demographics and comorbidities of participants

1. Italian study (N=2,908)
2. German study (N=2,028)
3. Canadian study (N=950)

**Supplementary Table 2**  Patient characteristics by 9p21.3 (rs4977574, rs2383207, and rs10738610) genotypes for severity of CAD

**Supplementary Table 3** Genotyping information of genotyped and imputed SNP in the Italian study (N=2,908)

**Supplementary Table 4**

1. Observed genotypes and allele counts (proportions) in the Italian study (N=2,908)
2. Observed genotypes and allele counts (proportions) in the German study (N=2,028)
3. Observed genotypes and allele counts (proportions) in the Canadian study (N=950)

**Supplementary Table 5** Association results derived from linear regression between severity of CAD and 9p21.3 SNPs with interaction term (T2D * SNP) performed in the Italian study (N=2,908)

**Supplementary Table 6** Association results of rs4977574, rs2383207, and rs10738610 with other quantifying scores for severity of CAD in the Italian (N=2,908) study

**Supplementary Table 7** Linkage disequilibrium (LD) among the 11 9p21.3 SNPs in the Italian study (N=2,908)

**Supplementary Table 1.** Demographics and comorbidity of participants

1. Italian study (N=2,908)

|  | Unaffected without severity of CAD | Affected with severity of CAD |
| --- | --- | --- |
| No. of patients | 1367 | 1541  [1VD (n=493); 2VD (n=582); 3VD (n=466)] |
| **Population demographics and comorbidities** | | |
| Gender |  |  |
| Male | 798 (58.4%) | 1157 (75.1%) |
| Age, years | 49.86 ± 13.35 | 63.4 ± 10.2 |
| Body mass index, kg/m^2^ | 27.1 ± 4.3 | 28.2 ± 13.1 |
| Family history of CAD | 19 (12.3%) | 378 (24.3%) |
| Arterial hypertension | 778 (56.9%) | 981 (63.7%) |
| Current smoking status | 365 (23.7%) | 727 (47.1%) |
| Diabetes Mellitus | 18 (1.3%) | 547 (35.5%) |
| Hypercholesterolemia | 48 (3.1%) | 868 (56.3%) |
| Use of Statins | 35 (2.3%) | 1220 (79.2%) |
| Previous MI | 4 (0.3%) | 708 (45.9%) |
| Left ventricular ejection fraction, % | 56.7 ± 8.9 | 55.1 ± 10.2 |
| eGFR, mL/min/1.73m^2^ | 71.8 ± 22.9 |  |
| Creatinine, mg/dl | 0.95 ± 0.2 | 1.17 ± 0.3 |
| **Plasma lipids profile** |  |  |
| TC, mg/dl | 194.8 ± 41.8 | 181.3 ± 46.3 |
| TG, mg/dl | 135.9 ± 66.4 | 145.2 ± 75.6 |
| LDL-C, mg/dl | 115.6 ± 38.8 | 103.9 ± 39.1 |
| HDL-C, mg/dl | 51.4 ± 13.2 | 47.1 ± 13.1 |
| **Stenosis characteristics** |  |  |
| Stenosis per target coronary vessel | |  |
| Proximal LAD | 0 (0%) | 1278 (82.9%) |
| LAD | 0 (0%) | 905 (58.7%) |
| LCx | 0 (0%) | 405 (26.3%) |
| LCA | 0 (0%) | 1281 (83.1%) |
| RCA | 0 (0%) | 250 (16.2%) |
| Stenosis per vessel location | |  |
| OST | 0 (0%) | 104 (6.7%) |
| PROX | 0 (0%) | 620 (40.2%) |
| MID | 0 (0%) | 666 (43.2%) |
| DIST | 0 (0%) | 151 (9.8%) |
| Modified ACC/AHA score (n=1541) | |  |
| Type A | 0 (0%) | 205 (13.3%) |
| Type B1 | 0 (0%) | 367 (23.8%) |
| Type B2 | 0 (0%) | 448 (29.1%) |
| Type C | 0 (0%) | 521 (33.8%) |
| Diameter stenosis, % |  | 86.4 ± 10.69 |
| Bifurcation stenosis | 16 (1.17%) | 326 (21.2%) |

Continuous values are expressed as mean ± standard deviation; categorical values are expressed as total number and as percentage of the total in the number of unaffected or affected with severity of CAD (in parenthesis)

**B.** German study (N=2,028)

|  | Unaffected without severity of CAD | Affected with severity of CAD |
| --- | --- | --- |
| No. of patients | 111 | 1917  [1VD (n=1691); 2VD (n=226)] |
| **Population demographics and comorbidities** | | |
| Male | 79 (71.2%) | 1506 (78.5%) |
| Age, years | 64.9 ± 10.8 | 66.3 ± 10.5 |
| Body mass index, kg/m^2^ | 27.9 ± 4.7 | 26.9 ± 3.9 |
| Family history of CAD | 36 (32.4%) | 767 (40%) |
| Arterial hypertension | 66 (59.5%) | 767 (40%) |
| Current smoker | 9 (8.1%) | 289 (15.1%) |
| Diabetes Mellitus | 29 (26.1%) | 527 (27.5%) |
| Hypercholesterolemia | 77 (69.4%) | 1353 (70.6%) |

Continuous values are expressed as mean ± standard deviation; categorical values are expressed as total number and as percentage of the total in the number of unaffected or affected with severity of CAD (in parenthesis)

**C.** Canadian study (N=1,714)

|  | Affected with severity of CAD;  **Early-onset (EO)**  [1VD + 2VD (n=610); 3VD (n=340)] |
| --- | --- |
| No. of patients | 950 |
| **Population demographics and comorbidities** | |
| Gender |  |
| Male | 673 (70.8%) |
| Age, years | 56.1 ± 9.6 |
| Body mass index, kg/m^2^ | 28.3 ± 5.2 |
| Smoking, ever | 734 (77.3%) |
| MI | 517 (54.4%) |
| Hypertension drugs | 523 (55.2%) |
| Cholesterol drugs | 839 (88.6%) |
| ASA | 856 (90.7%) |
| ACE inhibitor | 856 (90.7%) |
| **Plasma lipids profile** | |
| TC, mmol/L | 5.91 ± 1.4 |
| TG, mmol/L | 2.33 ± 1.6 |
| LDL-C, mmol/L | 3.73 ± 1.1 |
| HDL-C, mmol/L | 1.17 ± 0.52 |

Continuous values are expressed as mean ± standard deviation; categorical values are expressed as total number and as percentage of the total in the number of unaffected or affected with severity of CAD (in parenthesis)

**Supplementary Table 2.** Patient characteristics by 9p21.3 (rs4977574, rs2383207, and rs10738610) genotypes for severity of CAD

|  | rs4977574 | | |  | rs2383207 | | |  | rs10738610 | | |  |
| --- | --- | --- | --- | --- | --- | --- | --- | --- | --- | --- | --- | --- |
|  | 0 | 1 | 2 | *P* | 0 | 1 | 2 | *P* | 0 | 1 | 2 | *P* |
|  | (n=446) | (n=1330) | (n=1037) |  | (n=295) | (n=1049) | (n=986) |  |  |  |  |  |
| Male | 298 (66.8%) | 900 (67.7%) | 690 (66.5%) | 0.835 | 192 (65.1%) | 690 (65.8%) | 645 (65.4%) | 0.971 | 225 (66.8%) | 715 (65.9%) | 597 (65.2%) | 0.875 |
| Age, yrs | 57.5 ± 14.2 | 56.9 ± 13.8 | 56.6 ± 13.2 | 0.634 | 56.2 ± 14.2 | 55.9 ± 14.3 | 55.6 ± 13.7 | 0.803 | 57.2 ± 14 | 56.1 ± 14.2 | 55.1 ± 13.7 | 0.042 |
| BMI, kg/m2 | 27.7 ± 4 | 28.3 ± 14.9 | 27.5 ± 3.7 | 0.286 | 27.7 ± 3.6 | 28.5 ± 16.8 | 27.6 ± 3.8 | 0.302 | 27.9 ± 3.8 | 28.5 ± 16.5 | 27.5 ± 3.8 | 0.287 |
| Diabetes | 76 (17%) | 267 (20.1%) | 189 (18.2%) | 0.285 | 37 (12.5%) | 172 (16.4%) | 146 (14.8%) | 0.236 | 53 (15.7%) | 186 (17.1%) | 120 (13.1%) | 0.044 |
| Family history of CAD | 46 (10.3%) | 177 (13.3%) | 151 (14.6%) | 0.087 | 23 (7.8%) | 94 (9%) | 115 (11.7%) | 0.052 | 30 (8.9%) | 106 (9.8%) | 102 (11.1%) | 0.419 |
| Hypertension | 269 (62.1%) | 837 (62.9%) | 634 (61.1%) | 0.574 | 182 (63.9%) | 660 (64.8%) | 607 (63.3%) | 0.774 | 209 (64.3%) | 685 (65%) | 565 (63.4%) | 0.769 |
| Smoking | 158 (35.4%) | 510 (38.3%) | 367 (35.3%) | 0.273 | 104 (51.5%) | 381 (52.3%) | 331 (48.3%) | 0.307 | 122 (50%) | 406 (53.3%) | 293 (47.6%) | 0.106 |
| Previous MI | 92 (20.6%) | 325 (24.4%) | 251 (24.2%) | 0.232 | 50 (16.9%) | 202 (19.3%) | 187 (19%) | 0.660 | 64 (19%) | 221 (20.4%) | 159 (17.4%) | 0.237 |
| EF, % | 49.4±11.4 | 50.56±11.6 | 50.7±12.1 | 0.232 | 48.6 ± 11.5 | 48.7 ± 11.7 | 49.45 ± 12.2 | 0.414 | 48.8 ± 11.3 | 49.5 ± 11.5 | 48.8 ± 12.2 | 0.551 |
| eGFR, mL/min/1.73m^2^ | 69.9±19.5 | 70.9±18.3 | 71.4±18.7 | 0.501 | 71.6 ± 20.5 | 72 ± 17.9 | 72.1 ± 19.4 | 0.947 | 70.8 ± 20 | 72.2 ± 18.6 | 72.3 ± 18.8 | 0.564 |
| TC | 188±46.96 | 187.3±41.7 | 183.3±43.7 | 0.581 | 192.2 ± 48.7 | 190.4 ± 41.8 | 191.5 ± 41.8 | 0.817 | 190.4 ± 42.4 | 190.5 ± 41.5 | 190.5 ± 41.6 | 0.998 |
| LDL | 113.1±43.3 | 109.9±35.6 | 111.1±36.9 | 0.378 | 116 ± 45.7 | 112.6 ± 36.7 | 113.9 ± 35.9 | 0.495 | 114.1 ± 39 | 112.8 ± 36.3 | 113.1 ± 35.9 | 0.877 |
| HDL | 48.5±13.6 | 47.7±11.8 | 48.7±12.7 | 0.172 | 48.4 ± 13.4 | 48.2 ± 11.9 | 48.8 ± 12.2 | 0.620 | 48.4 ± 13.1 | 48.4 ± 12.1 | 48.9 ± 12.4 | 0.721 |
| TG | 144.7±73.1 | 140.6±68.9 | 140.9±72.4 | 0.603 | 150.1 ± 78.7 | 140.2 ± 68.5 | 141.1 ± 73.3 | 0.191 | 148.3 ± 76.6 | 140.9 ± 69.9 | 139.8 ± 73.7 | 0.267 |
| Stenosis per target coronary vessel | | |  |  |  |  |  |  |  |  |  |  |
| PLAD | 167 (37.4%) | 565 (42.5%) | 476 (45.9%) | 0.009 | 91 (30.8%) | 358 (34.1%) | 385 (39%) | 0.011 | 117 (34.7%) | 390 (35.9%) | 333 (36.4%) | 0.861 |
| LAD | 118 (26.5%) | 397 (29.8%) | 336 (32.4%) | 0.067 | 70 (23.7%) | 253 (24.1%) | 266 (27%) | 0.268 | 87 (25.8%) | 271 (25%) | 236 (25.8%) | 0.902 |
| CX | 53 (11.9%) | 183 (13.8%) | 153 (14.8%) | 0.338 | 24 (8.1%) | 114 (10.9%) | 133 (13.5%) | 0.025 | 33 (9.8%) | 129 (11.9%) | 110 (12%) | 0.518 |
| RCA | 31 (7%) | 116 (8.7%) | 91 (8.8%) | 0.458 | 18 (6.1%) | 72 (6.9%) | 60 (6.1%) | 0.750 | 21 (6.2%) | 75 (6.9%) | 56 (6.1%) | 0.756 |

Values are n (%) or mean ± SD unless otherwise noted. BMI=body mass index; CAD=coronary artery disease; MI=myocardial infarction; EF=ejection fraction; eGFR=estimated glomerular flirtation rate; TC=total cholesterol; LDL=low-density lipoprotein cholesterol; HDL=high density lipoprotein cholesterol; TG=triglycerides; PLAD=proximal left anterior descending artery; LAD=left anterior descending artery; CX; left circumflex artery; RCA=right coronary artery;

Supplementary Table 3. Genotyping information of genotyped and imputed SNPs in the Italian study (N=2,928)

|  |  |  |  |  |  | **Genotyping technique** | |  |  |  |
| --- | --- | --- | --- | --- | --- | --- | --- | --- | --- | --- |
| **SNP** | **Position** | **Alleles** | **MAF** | **Risk allele** | **Freq of risk allele** | **TaqMan**  **(n=1,696)** | **Illumina**  **Human1M-Duo**  **(n=1,212)** | **Imputed SNPs**  **(n=1,212)** | **Imputation quality (R^2^)** | **TRAIT** |
| rs7044859 | 22,008,781 | A/T | 0.463 | A | 0.538 | 1 | 1 |  |  |  |
| rs10965215 | 22,019,445 | A/G | 0.435 | A | 0.565 | 1 | 1 |  |  |  |
| rs564398 | 22,019,547 | C/T | 0.301 | T | 0.699 | 1 | 1 |  |  | T2D |
| rs7865618 | 22,021,005 | A/G | 0.304 | A | 0.590 | 1 | 1 |  |  | CHD |
| rs10116277 | 22,071,397 | G/T | 0.390 | T | 0.610 | 1 |  | 1 | 0.946 | CAD |
| rs4977574 | 22,088,574 | A/G | 0.398 | G | 0.602 | 1 | 1 |  |  | CAD |
| rs2383207 | 22,105,959 | A/G | 0.360 | G | 0.640 | 1 | 1 |  |  | CAD |
| rs10738610 | 22,113,766 | A/C | 0.391 | C | 0.609 | 1 |  | 1 | 0.961 | CAD |
| rs10757278 | 22,114,477 | A/G | 0.422 | G | 0.578 | 1 |  | 1 | 0.990 | CAD |
| rs1333049 | 22,115,503 | C/G | 0.420 | C | 0.580 | 1 |  | 1 | 0.991 | CAD |
| rs10811661 | 22,124,094 | C/T | 0.206 | T | 0.795 | 1 | 1 |  |  | T2D |

MAF = Minor allele frequency; T2D, Type 2 diabetes; CHD, coronary heart disease; CAD, coronary artery disease;

Supplementary Table 4.

A. Observed genotypes and allele counts (proportions) in the Italian study (N=2,908)

| **SNP** | **Position** | **N** | **Risk allele** | **Genotype Count (Proportion)** | | | **Allele Count (Proportion)** | | **HWE *P*** |
| --- | --- | --- | --- | --- | --- | --- | --- | --- | --- |
| rs7044859 | 22,008,781 | 2761 | A | A/A | A/T | T/T | A | T |  |
|  |  |  |  | 791 (0.29) | 1383 (0.50) | 587 (0.210) | 2965 (0.54) | 2557 (0.46) | 0.731 |
| rs10965215 | 22,019,445 | 2859 | A | A/A | A/G | G/G | A | G |  |
|  |  |  |  | 923 (0.32) | 1383 (0.48) | 553 (0.19) | 3229 (0.56) | 2489 (0.44) | 0.402 |
| rs564398 | 22,019,547 | 2857 | T | T/T | T/C | C/C | T | C |  |
|  |  |  |  | 1421 (0.50) | 1180 (0.41) | 256 (0.09) | 4022 (0.70) | 1692 (0.30) | 0.621 |
| rs7865618 | 22,021,005 | 2856 | A | A/A | A/G | G/G | A | G |  |
|  |  |  |  | 1408 (0.49) | 1185 (0.41) | 263 (0.09) | 4001 (0.70) | 1711 (0.30) | 0.562 |
| rs10116277 | 22,071,397 | 2307 | T | T/T | T/G | G/G | T | G |  |
|  |  |  |  | 874 (0.38) | 1084 (0.47) | 349 (0.15) | 2832 (0.61) | 1782 (0.39) | 0.661 |
| rs4977574 | 22,088,574 | 2813 | G | G/G | G/A | A/A | G | A |  |
|  |  |  |  | 1037 (0.37) | 1330 (0.47) | 446 (0.16) | 3404 (0.61) | 2222 (0.39) | 0.581 |
| rs2383207 | 22,105,959 | 2330 | G | G/G | G/A | A/A | G | A |  |
|  |  |  |  | 986 (0.42) | 1049 (0.45) | 295 (0.13) | 3021 (0.65) | 1639 (0.35) | 0.555 |
| rs10738610 | 22,113,766 | 2337 | C | C/C | C/A | A/A | C | A |  |
|  |  |  |  | 915 (0.39) | 1085 (0.46) | 337 (0.14) | 2915 (0.62) | 1759 (0.38) | 0.597 |
| rs10757278 | 22,114,477 | 2872 | G | G/G | G/A | A/A | G | A |  |
|  |  |  |  | 1007 (0.35) | 1395 (0.49) | 470 (0.16) | 3409 (0.59) | 2335 (0.41) | 0.757 |
| rs1333049 | 22,115,503 | 2869 | C | C/C | C/G | G/G | C | G |  |
|  |  |  |  | 987 (0.34) | 1408 (0.49) | 474 (0.17) | 3382 (0.59) | 2356 (0.41) | 0.464 |
| rs10811661 | 22,124,094 | 2874 | T | T/T | T/C | C/C | T | C |  |
|  |  |  |  | 1893 (0.66) | 883 (0.31) | 98 (0.03) | 4669 (0.81) | 1049 (0.19) | 0.714 |

HWE, Hardy Weinberg Equilibrium;

**B.** Observed genotypes and allele counts (proportions) in the German (N=2,028) study

| **SNP** | **Position** | **N** | **Risk allele** | **Genotype Count (Proportion)** | | | **Allele Count (Proportion)** | | **HWE *P*** |
| --- | --- | --- | --- | --- | --- | --- | --- | --- | --- |
| rs1333049 | 22,115,503 | 2,028 | C | C/C | C/G | G/G | C | G |  |
|  |  |  |  | 526 (0.26) | 1018 (0.50) | 484 (0.24) | 2070 (0.51) | 1986 (0.49) | 0.859 |

HWE, Hardy Weinberg Equilibrium;

**C.** Observed genotypes and allele counts (proportions) in the Canadian study (N=950)

| **SNP** | **Position** | **N** | **Risk allele** | **Genotype Count (Proportion)** | | | **Allele Count (Proportion)** | | **HWE *P*** |
| --- | --- | --- | --- | --- | --- | --- | --- | --- | --- |
| rs4977574 | 22,088,574 | 953 | G | G/G | G/A | A/A | G | A |  |
|  |  |  |  | 294 (0.31) | 479 (0.50) | 177 (0.19) | 1073 (0.56) | 833 (0.44) | 0.560 |

HWE, Hardy Weinberg Equilibrium;

**Supplementary Table 5.** Association results derived from linear regression between severity of CAD and 9p21.3 SNPs with interaction term (T2D * SNP) performed in the Italian study (N=2,908)

| SNP | Position | Beta_int_ | s.e._int_ | *P_int_* |
| --- | --- | --- | --- | --- |
| rs7044859 | 22,008,781 | -0.023 | 0.063 | 7.14E-01 |
| rs10965215 | 22,019,445 | -0.026 | 0.062 | 6.82E-01 |
| rs564398 | 22,019,547 | -0.021 | 0.069 | 7.66E-01 |
| rs10116277 | 22,071,397 | 0.108 | 0.065 | 9.83E-02 |
| rs4977574 | 22,088,574 | 0.107 | 0.064 | 9.53E-02 |
| rs2383207 | 22,105,959 | 0.128 | 0.066 | 5.33E-02 |
| rs10738610 | 22,113,766 | 0.132 | 0.065 | **4.28E-02** |
| rs10757278 | 22,114,477 | 0.075 | 0.064 | 2.44E-01 |
| rs1333049 | 22,115,503 | -0.030 | 0.064 | 6.44E-01 |
| rs10811661 | 22,124,094 | 0.002 | 0.082 | 9.85E-01 |

*Beta_int_ is the interaction effect of T2D*SNP derived from the model: severity of CAD ~ age + sex + T2D + T2D*SNP; *P_int_* denotes nominal significance of <0.05 for the interaction term;

**Supplementary Table 6.** Association results of rs4977574, rs2383207, and rs10738610 with other quantifying scores for severity of CAD in the Italian (N=2,908) study

|  | Beta | s.e. | *P* | *P_emp_* |
| --- | --- | --- | --- | --- |
| **Gensini score** |  |  |  |  |
| rs4977574-G | 0.048 | 0.365 | 8.96E-01 | 9.92E-01 |
| rs2383207-G | 0.315 | 0.437 | 4.71E-01 | 8.51E-01 |
| rs10738610-C | 0.286 | 0.422 | 4.98E-01 | 9.97E-01 |
| **Duke score** |  |  |  |  |
| rs4977574-G | 2.025 | 0.692 | **3.47E-03** | **1.81E-02** |
| rs2383207-G | 2.598 | 0.885 | **3.41E-03** | **2.94E-02** |
| rs10738610-C | 2.816 | 0.854 | **1.00E-03** | 6.14E-02 |
| **Dahlen score** |  |  |  |  |
| rs4977574-G | 10.656 | 2.976 | **3.53E-04** | **2.70E-03** |
| rs2383207-G | 12.561 | 3.783 | **9.27E-04** | **1.18E-02** |
| rs10738610-C | 13.487 | 3.645 | **2.26E-04** | **2.86E-02** |
| **Diameter stenosis, %** | |  |  |  |
| rs4977574-G | 3.421 | 0.946 | **3.06E-04** | **1.10E-03** |
| rs2383207-G | 3.467 | 1.242 | **5.34E-03** | **4.59E-02** |
| rs10738610-C | 4.264 | 1.198 | **3.88E-04** | 6.44E-02 |

*Beta is the genetic effect derived from the model: score for severity of CAD ~ age + sex + T2D; Boldface *P* denotes nominal significance of <0.05; *P_emp_* denotes empirical significance after 10,000 permutations;

**Supplementary Table 7.** Linkage disequilibrium (LD, *r^2^*) among the 11 9p21.3 variants in the Italian study (N=2,908)

| *r^2^* | rs7044859 | rs10965215 | rs564398 | rs7865618 | rs10116277 | rs4977574 | rs2383207 | rs10738610 | rs10757278 | rs1333049 | rs10811661 |
| --- | --- | --- | --- | --- | --- | --- | --- | --- | --- | --- | --- |
| rs7044859 | 1.00 | 0.90 | 0.70 | 0.70 | 0.46 | 0.42 | 0.43 | 0.41 | 0.36 | 0.35 | 0.06 |
| rs10965215 |  | 1.00 | 0.74 | 0.74 | 0.50 | 0.47 | 0.46 | 0.45 | 0.41 | 0.40 | 0.06 |
| rs564398 |  |  | 1.00 | 0.92 | 0.24 | 0.24 | 0.18 | 0.21 | 0.22 | 0.21 | 0.00 |
| rs7865618 |  |  |  | 1.00 | 0.28 | 0.28 | 0.22 | 0.25 | 0.27 | 0.26 | 0.00 |
| rs10116277 |  |  |  |  | 1.00 | 1.00 | 0.93 | 0.96 | 0.87 | 0.89 | 0.00 |
| rs4977574 |  |  |  |  |  | 1.00 | 0.93 | 0.96 | 0.87 | 0.89 | 0.00 |
| rs2383207 |  |  |  |  |  |  | 1.00 | 0.96 | 0.88 | 0.89 | 0.00 |
| rs10738610 |  |  |  |  |  |  |  | 1.00 | 0.92 | 0.93 | 0.00 |
| rs10757278 |  |  |  |  |  |  |  |  | 1.00 | 1.00 | 0.00 |
| rs1333049 |  |  |  |  |  |  |  |  |  | 1.00 | 0.00 |
| rs10811661 |  |  |  |  |  |  |  |  |  |  | 1.00 |
